# Immunohistochemistry Study of Antimicrobial Peptides as a Future Diagnostic and Prognostic Tool for Periprosthetic Joint Infections

**DOI:** 10.7759/cureus.69629

**Published:** 2024-09-18

**Authors:** Emanuel-Cristian Sandu, Bogdan Serban, Sergiu Iordache, Adrian Cursaru, Mihai Aurel Costache, Adrian Dumitru, Catalin Cirstoiu

**Affiliations:** 1 Orthopedics and Traumatology, "Carol Davila" Faculty of Medicine, Bucharest, ROU; 2 Orthopedics and Traumatology, University Emergency Hospital, Bucharest, ROU; 3 Pathology, "Carol Davila" Faculty of Medicine, Bucharest, ROU; 4 Pathology, University Emergency Hospital, Bucharest, ROU

**Keywords:** antimicrobial peptides (amp), cathelicidin, hbd-3, human beta defensin 3, immunohistochemistry staining, ll-37, periprosthetic joint infection (pji), pji diagnosis

## Abstract

Periprosthetic joint infection (PJI) is a reputable complication of arthroplasty surgery. Septic loosening is an implant biofilm-related infection with different characteristics and treatment than aseptic loosening. Misdiagnosing PJI results in choosing an inappropriate treatment and, in most cases, failure to achieve asepsis. The worldwide increase of arthroplasty surgeries forces us to research more accurate ways to detect PJIs earlier, cheaper, and faster. In the current study, we investigated 52 arthroplasty revision surgeries (septic and aseptic) and, using immunohistochemistry staining of periprosthetic tissue, successfully demonstrated an important increase in antimicrobial peptides human β defensin-3 (HBD-3) and cathelicidin (LL-37) in the PJI group. Furthermore, we observed that patients with a positive LL-37 stain were associated with a more reserved prognosis at one-year follow-up. These promising results suggest that antimicrobial peptides HBD-3 and LL-37 could be used as future biomarkers for PJI detection.

## Introduction

Periprosthetic joint infection (PJI) is one of the most feared complications after joint replacement surgery. Even though the incidence is as low as 1% [1], it can go up to 5% with added risk factors like cigarette smoking, diabetes, overweight, and more, going even higher after each revision surgery. With the increase in primary arthroplasty surgeries, we expect an increase in the total number of revisions as well in the following years [2]. If present, PJI is associated with important patient morbidity, difficult treatment, long hospital stays, and financial burden of the medical care. Even with the novel advancement of technology, great efforts are still made to develop a reliable and accurate diagnostic test and treatment protocol. Nowadays, diagnosis is made using multiple criteria proposed by the major orthopedic organizations like the European Bone and Joint Infection Society (EBJIS), the Infectious Diseases Society of America (IDSA), and the Musculoskeletal Infection Society (MSIS) [3]. The key for a successful treatment is fast and accurate diagnostic of the infection.

More recent studies have explored the presence and role of antimicrobial peptides (AMP) in PJI detection and pathophysiology [4]. AMP are a class of small peptides that exist in nature and are an important part of the innate immune system [5]. The presence of these molecules is essential for defense against invasive bacterial, viral, or fungal infections. Besides their direct antimicrobial function, AMPs have multiple roles in mediating innate and adaptive immunity such as neutrophil chemotaxis, mast cell recruitment, endotoxin neutralization, and wound healing. Amphipathic properties have a vital role in the activity of these peptides [6]. This property allows AMP to integrate into the cell membrane or pass through into the cytosol leading to apoptosis of the targeted cell. There are three main groups of AMPs in humans. The first group is the defensins, which are cationic peptides subdivided in two classes: α-defensins and β-defensins. The second group is cathelicidins, with only one AMP present in humans, LL-37. The third group is the family of histatins, mainly present in human saliva [7].

Knowing the importance and mechanism of AMPs in bacterial infections, more studies have been published about their role in PJI. The most relevant AMPs were α-defensins 1-3, human β defensins 2 and 3 (HBD-2, HBD-3), and the cathelicidin LL-37. Some of the results are discussed further in the article. A successful diagnostic method emerged in the form of α defensin detection in the synovial fluid using a test kit (Synovasure) or ELISA technique [8], which was approved as a PJI diagnostic criteria by MSIS.

Histopathological grading of the periprosthetic membrane proposed by Morawietz et al. [9] is still considered one of the most important criteria in diagnosing PJI. Although identifying inflammatory cells in the examined tissue yields good results in diagnosing PJI, improvements could be made to increase accuracy. Using further immunohistochemistry (IHC) analysis of the periprosthetic tissue, we tried to facilitate the histological diagnosis of PJI, hoping to achieve better outcomes for patients undergoing arthroplasty revision surgery. HBD-3 and cathelicidin LL-37 were the studied biomarkers.

## Materials and methods

Patients and study design

Over the four-year study period (2020-2024) at the University Emergency Hospital, Bucharest, we included 52 of 165 hip and knee arthroplasty revision cases to obtain relevant and novel data regarding the diagnosis and prognosis of PJI. For this prospective study (diagnostic level II), we enrolled consecutive patients who met our inclusion criteria (Table 1).

**Table 1:** Inclusion and exclusion criteria for the study

Inclusion criteria	Exclusion criteria
Hip or knee revision surgery of primary arthroplasty	Mechanical complications of prosthesis like periprosthetic fracture and prosthesis luxation
Age above 18 years	Immediate revision surgery required because of component malposition or unstable fixation
Septic or aseptic loosening of hip or knee prosthesis	Follow-up less than one year
	Partial joint replacement
	Septic or aseptic loosening cases that had major contraindications of revision surgery
	The patient refused informed consent

Using the updated 2018 MSIS criteria, patients were divided into two groups: aseptic loosening (AL, n = 29) and periprosthetic joint infection (PJI, n = 23). For an early diagnosis, we used clinical findings like sinus tract formation, purulence at the site of prosthesis, radiographs, and the standard paraclinical findings using serum C-reactive protein (CRP), white blood cell count (WBC), erythrocyte sedimentation rate (ESR), and joint aspiration. Pathological microorganisms were identified using standard bacterial cultures of synovial fluid, tissue samples obtained during the surgery, or sonication fluid of the explanted prosthesis. Furthermore, histopathological grading of the periprosthetic membrane was addressed using the Morawietz et al. algorithm [9]. The most important demographic, clinical, and laboratory data of the studied patients are shown in Table 2.

**Table 2:** Demographic, clinical, and laboratory data of the studied population CRP: C reactive protein; ESR: Erythrocyte sedimentation rate; WBC: White blood cell; DAIR: Debridement, antibiotics, and implant retention.

Variables	PJI (n = 23)	AL (n = 29)	P-value
Median age (years)	68.36 (34-90)	65.28 (46-87)	
Female gender	9	17	
*Prosthetic joint*			
Hip	18	28	
Knee	5	1	
Diabetes	9	3	0.014
Smoking	15	4	<0.001
Serum CRP mean	52.14 mg/L (1.9-293.7)	8.26 mg/L (0.2-45.30)	<0.001
Serum ESR mean	52.43 mm/h (5-89)	18.29 mm/h (2-48)	<0.001
Serum WBC mean	8.70 × 10³/uL (4.6-14.8)	7.35 × 10³/uL (4-12.4)	0.068
Serum fibrinogen mean	503.17 mg/dL (191-793)	363.24 mg/dL (254-488)	<0.001
*Surgical approach*			
DAIR	5	0	
One step revision	2	27	
Two steps revision	16	2	
Positive preoperatory culture	15	0	
Positive tissue culture	12	3 (False positive)	

Tissue preparation

During revision surgery, the operating team collected multiple periprosthetic tissue biopsies for bacteriological cultures and histology. One additional biopsy of periprosthetic tissue that was in direct contact with the components of the prosthesis was harvested for the study. The biopsy was immediately fixated using 4% formaldehyde solution and calcium carbonate for pH neutralization. The mean time for fixation was 12 hours depending on the sample size and ambient temperature. The process was followed by dehydration of the pieces using multiple alcohol concentrations and clarification with toluene. The paraffin-wax embedding process of the samples was performed at 58°C-60°C, obtaining the paraffin blocks that were labeled with the patient's information. Using a rotary microtome, 5-µm thick sections were obtained that were assembled on microscope slides.

Immunohistochemistry assay

The process was performed using a fully automated slide staining system (ONCORE Pro, Biocare Medical, Pacheco, CA) that has the capabilities of slide baking, deparaffinization, antigen retrieval, counterstaining, and antibody detection when used for IHC. After deparaffinization, rehydration, and endogenous peroxidase blocking using phosphate buffer saline (PBS, Biocare Medical, Pacheco, CA) and 1% hydrogen peroxide, unmasking of antigenic epitopes for antigen retrieval was performed by exposure to heat at 101°C for both HBD3 and LL37. IHC staining was done using third-party primary antibodies against human HBD-3 (rabbit, polyclonal, ABIN6942250, Antibodies-online GmbH, Aachen, Germany) and LL-37 (rabbit, polyclonal, ABIN3022306, Antibodies-online GmbH, Aachen, Germany). The samples were incubated at 27°C with a pH of 7.2 for 59 minutes using a dilution of 1:100 for LL-37 and 1:200 for HBD-3 followed by PBS washing. Detection was performed using the Biocare ONCORE Pro system with anti-rabbit biotin-free detection labeled with horseradish peroxidase (HRP, Biocare Medical, Pacheco, CA) and another PBS washing afterward. 3,3’-Diaminobenzidine (DAB, Biocare Medical, Pacheco, CA) was used as a chromogen, producing a brown precipitate. Nuclear counterstaining was done using Tacha’s Automated Hematoxylin (Biocare Medical, Pacheco, CA) to increase the contrast.

Semiquantitative IHC analysis

The resulting slides were analyzed under microscopy with x10, x20, and x40 magnification. From three to five slides, the most representative regions were selected and evaluated by our investigator and graded using the immune reactive score (IRS) proposed by Remmele and Stegner [10]. IRS is obtained by multiplying the stained cells' score with their staining intensity score. The number of stained cells is quantified by percentage, and equivalent scores are assigned. The staining intensity is divided into four groups. The detailed algorithm is exposed in Table 3.

**Table 3:** Immune reactive score

Percentage of stained cells	Staining intensity	Stained cells score/Intensity score
0%	Negative	0
<10%	Weak	1
10%-50%	Intermediate	2
51%-80%	Strong	3
>80%	-	4
*Final score (Stained cell score × Intensity score)*		
0-1	Negative	
2-3	Positive-weak	
4-8	Positive-moderate	
9-12	Positive-strong	

With a score ranging from 0 to 12, we considered 0 and 1 as negative results and 2-12 as positive results to interpret the IHC staining in both PJI and AL groups.

Statistical analysis

Data from the two groups was gathered using Microsoft Excel 2019 software (Microsoft Corp., Redmond, Washington), and statistical analyses were performed using IBM SPSS Statistics version 21.0 (IBM Corp., Armonk, NY) and MedCalc® Statistical Software version 22.026 (MedCalc, West Flanders, Belgium). Hypothesis testing for group differences was performed using the Mann-Whitney U test, Wilcoxon test, and Chi-square test. A 95% confidence interval was used with a maximum accepted error deviation of ±5%. P-values less than 0.05 were considered statistically relevant.The accuracy of antimicrobial peptide IHC detection using HBD-3 and LL-37 antibodies in the periprosthetic tissue as a diagnostic tool for PJI was determined by the area under the curve (AUC) value from a receiver operating characteristic (ROC) curve analysis. An AUC value of 0.5 was correlated with a test that has no diagnostic strength, while an AUC value greater than 0.9 (maximum value: 1) showed an excellent diagnostic strength with high sensitivity and high specificity of the test.

## Results

As stated earlier, patients were categorized as either PJI or AL based on MSIS criteria. The most relevant parameters showing an important statistical difference between the two groups were serum CRP level and ESR value. With a mean of 52.14 mg/L (1.9-293.7) for PJI and 8.26 mg/L (0.2-45.30) for AL, CRP levels were almost seven times higher in the PJI group (p < 0.001), and ESR levels were almost three times higher in the PJI group than AL, with a mean of 52.43 mm/h (5-89) and 18.29 mm/h (2-48), respectively (p < 0.001). Cigarette smoking and diabetes were more frequent in the PJI group. No significant differences were noted in the serum WBC levels between the groups (p = 0.068).

**Figure 1:**
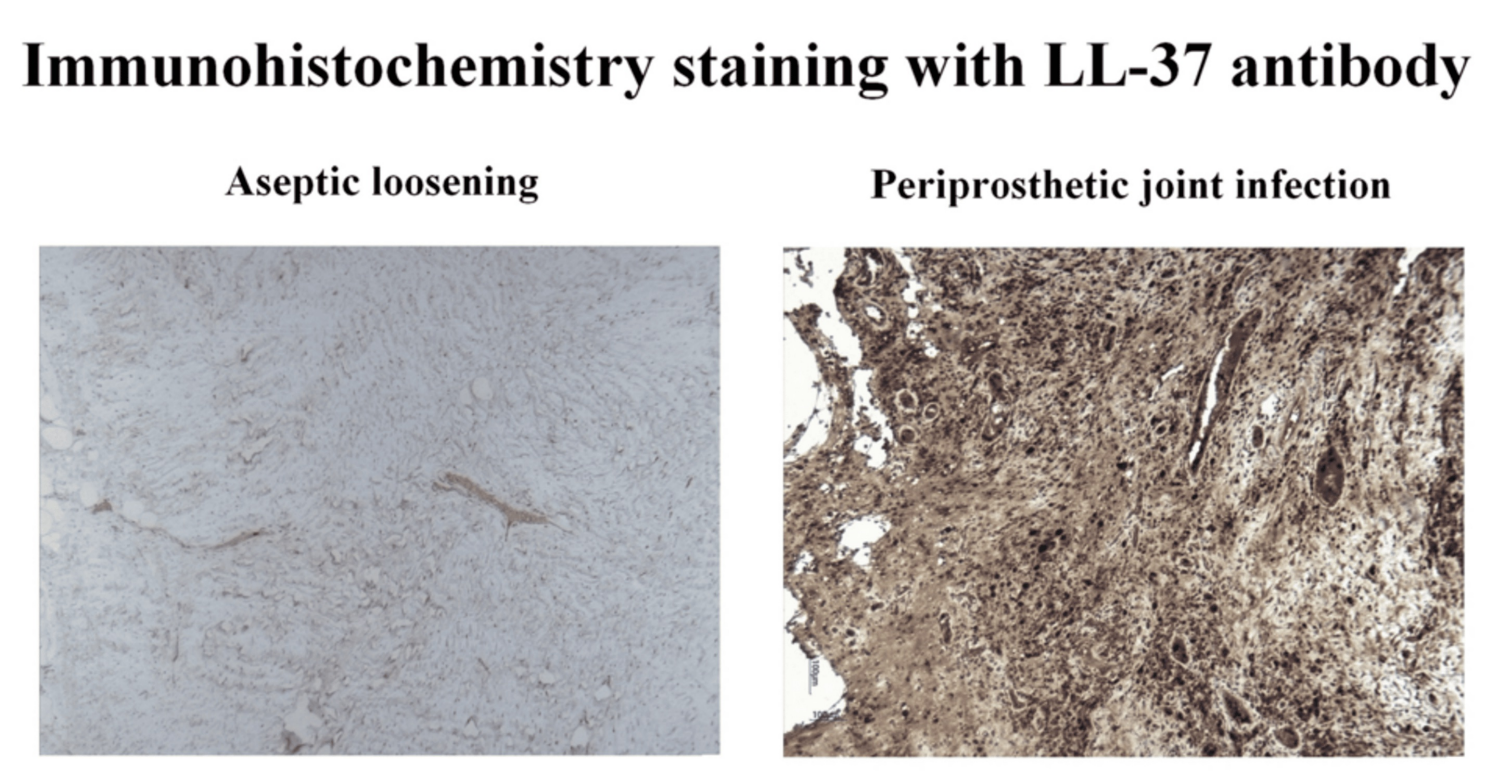
Immunohistochemistry LL-37 analysis of periprosthetic tissue. Representative stained sections of the IHC study using LL-37 antibody and DAB chromogen showed a significant increase of brown dying in the PJI group. LL-37: Cathelicidin; IHC: Immunohistochemistry; DAB: 3,3’-Diaminobenzidine; PJI: Periprosthetic joint infection.

**Figure 2:**
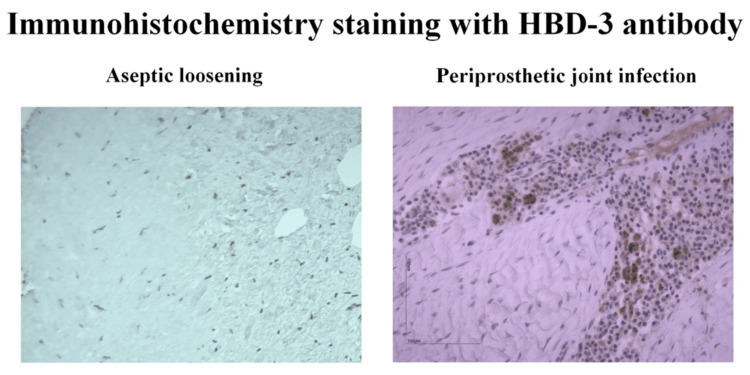
Immunohistochemistry HBD-3 analysis of periprosthetic tissue. Representative stained sections of the IHC study using HBD-3 antibody and DAB chromogen showed an increase of brown dying in the PJI group. HBD-3: Human β defensin 3; IHC: Immunohistochemistry; DAB: 3,3’-Diaminobenzidine; PJI: Periprosthetic joint infection.

Immunohistochemistry analysis of the collected periprosthetic tissue samples was conducted on the resulting slides from the aforementioned automated process. Representative images are presented in Figures 1, 2 to show the clearly visible increase in chromogen dying (DAB-brown) in the slides from patients with PJI compared to those in the AL group.

**Figure 3:**
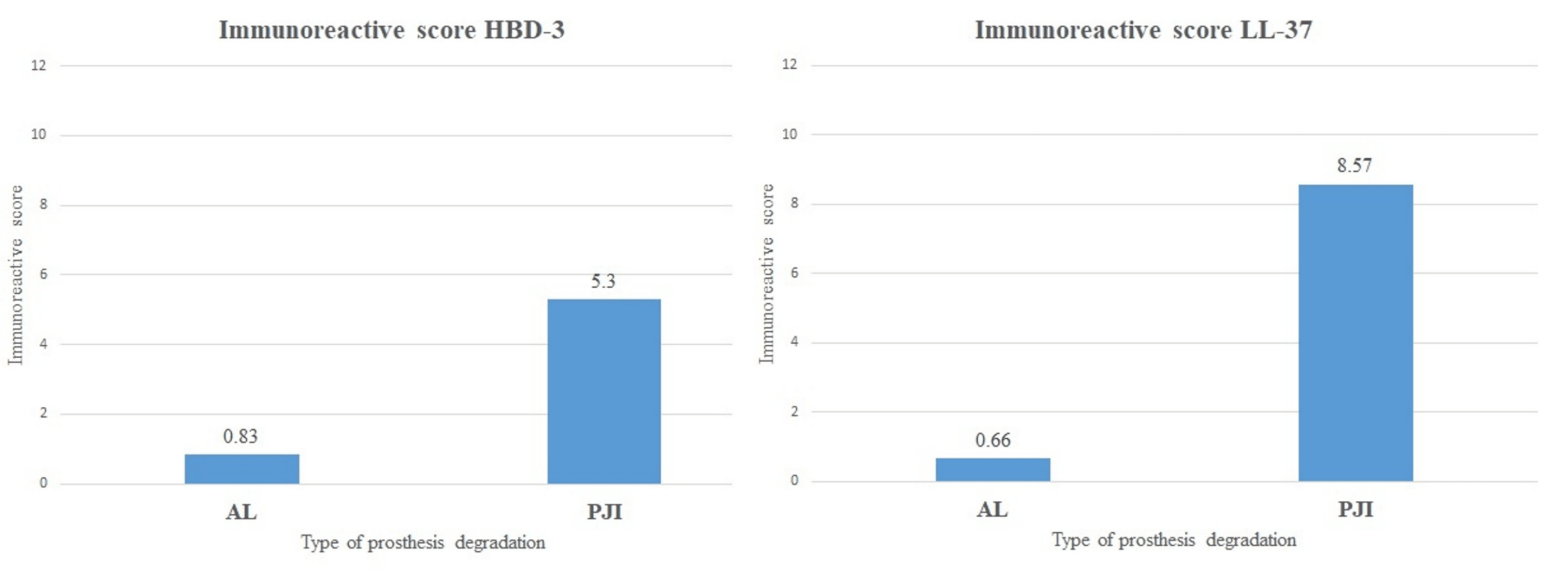
Immune reactive scores of human β defensin 3 (HBD-3) and cathelicidin (LL-37) AL: Aseptic loosening; PJI: Periprosthetic joint infection.

Furthermore, IHC staining IRS was used as a semiquantitative analysis, confirming an important difference between the two groups regarding HBD-3 and LL-37. The mean of IRS results are presented in Figure 3, showing an increase of almost 13 times in PJI versus AL when LL-37 was used and almost six times when HBD-3 was tested (p < 0.001). Accuracy of PJI detection using IHC staining of periprosthetic tissue with LL-37 and HBD-3 antibodies was assessed with the help of AUC in an ROC curve analysis, showing excellent diagnostic potential with a value of 0.987 for LL-37 and 0.925 for HBD-3 (p < 0.001).

Furthermore, we investigated whether there was significant statistical data regarding the evolution of the patients and IHC results, promoting the presence of LL-37 in periprosthetic tissue not only as a diagnostic tool but also as a prognosis factor. LL-37 was chosen because of the higher difference in IRS between the two groups.

Data for LL-37 showed that 83.33% of the patients with unfavorable evolution at one year had positive LL37 (n = 10) and 16.67% had negative LL37 (n = 2). In the favorable evolution group, 27.78% of the patients had positive LL37 (n = 10) and 72.22% had negative LL37 (n = 26). Four patients were deceased at one year, and 75% (n = 3) of them had positive LL37 on IHC samples. The results are also presented in tabular form for easier interpretation in Table 4. Using a Chi-square test, we observed that there is a moderate tendency of unfavorable evolution at one year among the patients with positive IHC stain for LL37 (p = 0.002).

**Table 4:** Outcome of the patients one year after surgery according to the immunohistochemistry stain LL-37: Cathelicidin.

Immunohistochemistry stain	Favorable evolution at 1 year	Unfavorable evolution at 1 year	Deceased patients
*Positive *LL-37 stain	27.78% (n = 10)	83.33% (n = 10)	75% (n = 3)
*Negative *LL-37 stain	72.22% (n = 26)	16.67% (n = 2)	25% (n = 1)

## Discussion

Prosthesis loosening is an important complication of joint arthroplasty with a great impact on the patient's health and the healthcare system. While revision for AL has a favorable outcome in most cases with successful treatment being achieved in one surgery, septic loosening or PJI is a far more demanding condition, involving a greater morbidity level for the patient, multiple interventions needed to eradicate the infectious process, long hospital stays, and tremendous financial resources being spent [11].

The number one challenge of this pathology is still represented by diagnostic inaccuracy, mostly in cases of chronic low-grade PJI caused by bacteria with lower virulence [12]. Fast and accurate identification of the pathogen bacteria is essential for the success of the treatment. Most often method used is standard bacteriological culture. Unfortunately, the sensitivity of this type of technique to diagnose PJI remains relatively low [13] as evidenced in our study group as well. The preoperative cultures from synovial fluid managed to identify only 15 out of 23 PJIs. Additionally, the cultures inoculated with the harvested periprosthetic tissue samples successfully identified the pathogens in only 12 cases. The decrease in sensitivity of the second type of culture is mainly explained by the initiation of antibiotic therapy, causing an increase in false-negative findings [14]. Identification of pathogen bacteria was performed with greater success by inoculating bacteriological cultures with sonication fluid obtained after sonication of explanted prosthesis components.

An important increase in the serum CRP and ESR levels was observed in the septic group, which remained the most commonly used biomarkers for PJI screening. Results can be modified by age or polycythemia (in the case of ESR), systemic inflammation such as rheumatological diseases, or other infections; therefore, a negative finding cannot rule out a PJI [15].

The first ready-to-use test accepted for clinical use regarding AMP detection in arthroplasty revision surgery is the lateral flow test for α-defensin. Detection of α-defensin in synovial fluid using the lateral flow test or ELISA technique is considered a minor criterion in the updated MSIS diagnostic protocol, revealing a possibility of infection. More recent studies regarding the lateral flow test showed a lower sensitivity than anticipated [16].

The detection of AMP as an ancillary test for PJI diagnosis was furthermore investigated, and one study conducted by Banke et al. in 2020 showed increased levels of HBD-3 and LL37 for the first time in the synovial membrane of patients with septic loosening [17]. The targeted group included only patients with coagulase-negative *Staphylococci *(ConS) infections, considering the pathogen was related to low-grade infections. In a previous study by Paulsen et al., using polymerase chain reaction (PCR), the presence of HBD-3 and LL-37 in pyogenic arthritis and the absence of those biomarkers in the healthy synovial membrane were confirmed [18].

Acknowledging the information from those studies, we expanded the use of AMP as a tool for PJI identification to a slightly larger group and more diverse infectious pathogens such as *Staphylococcus aureus*, ConS, *Pseudomonas Spp*, *Klebsiella pneumonia, *and more. We managed to identify the presence of HBD-3 and LL-37 AMP not only in the synovial membrane but in periprosthetic tissue as well, the latest being the targeted tissue for the IHC staining in our study. One advantage of using periprosthetic tissue as a sample is the availability of the product in 100% of the cases, whereas synovial fluid can sometimes be scarce and insufficient for sampling.

Despite the evolution of more modern detection systems like PCR, next-generation sequencing (NGS), and matrix-assisted laser desorption ionization time-of-flight mass spectrometry (MALDI-TOF-MS), an alternative reliable and cheap biomarker for PJI detection is still researched [19]. Using IRS for IHC staining with HBD-3 and LL-37 antibodies, we managed to reinforce the results from previous studies, showing an important increase of AMPs in periprosthetic tissue in the PJI group. Also, results did not appear to be influenced by the administration of antibiotics, with sensitivity remaining almost constant throughout the group. Results could be obtained on the first or second day after revision surgery and could lead to further and more extensive investigations into infectious pathology.

With AUC results showing excellent diagnostic strength of the test, a positive IHC stain of HBD-3 and LL-37 AMP was almost exclusively associated with PJI. Knowing this information and the fact that revision surgery for PJI is associated with poorer outcomes, such as higher morbidity, higher mortality, and worse functional outcomes, compared with aseptic revision [20], we can further understand why patients with positive results had more unfavorable outcomes one year after revision surgery.A longer follow-up period is necessary to obtain more accurate data.

Several points need to be addressed regarding the current study. The uneven distribution of the involved joints (hip or knee) does not represent the current data on arthroplasty revision surgery, which shows an almost equal incidence of PJI in both knee and hip [1]. It is a result of our medical care staff's surgical abilities combined with the lower availability of knee revision implants in our clinic. Another limitation of the study is the relatively small group of patients; future studies are needed to strengthen the researched hypothesis.

## Conclusions

In this study, we demonstrated that the presence of AMP such as HBD-3 and LL-37 in periprosthetic tissue is closely related to PJI. Immunohistochemistry semiquantitative study of the tissue samples, being easy to perform, accessible, and cost-effective, showed promising results in identifying PJI. Furthermore, positive LL-37 staining was associated with more unfavorable outcomes at the one-year follow-up.

With advancements in proteomics and molecular biology, even faster and more accurate diagnostic tests could be developed for detecting PJIs using AMP. The ultimate goal is to implement these studied biomarkers as real-time treatment-deciding factors for arthroplasty revision surgery.
